# Magnetic Electrospun Polycaprolactone (PCL) Fibers Modified with Oleic Acid-Coated Iron Oxide Nanoparticles

**DOI:** 10.1021/acsomega.6c07387

**Published:** 2026-10-07

**Authors:** Angie N. Morales, Danay M. Jaime, Guilherme M. O. Barra

**Affiliations:** Department of Mechanical Engineering, Federal University of Santa Catarina (UFSC), 88040-970 Florianópolis, SC, Brazil

## Abstract

Magnetic polycaprolactone (PCL) fibers containing oleic acid-coated iron oxide nanoparticles (IONPs/OA) were produced by electrospinning. First, a systematic study was carried out to determine the optimal processing parameters, establishing a 20% (w/v) PCL concentration, an applied voltage of 20 kV, a needle-to-collector distance of 22 cm, and a flow rate of 2 mL/h as the most suitable parameters. Homogeneous neat PCL fibers with an average diameter of 543 ± 88 nm were obtained under these conditions. Subsequently, different IONPs/OA concentrations (5, 10, and 20 wt %) were incorporated, leading to a progressive increase in fiber diameter, with a maximum of 961 ± 201 nm for the 20 wt % formulation. Magnetic measurements confirmed superparamagnetic behavior at room temperature for all composite fiber samples, with a maximum saturation magnetization (*M*_s_) of 6.1 ± 0.1 emu/g for the 20 wt % formulation. Thermogravimetric analysis revealed that the incorporation of IONPs/OA into the fibers decreased the degradation temperature by approximately 63 °C compared to neat PCL. Finally, water contact angle (WCA) measurements revealed a progressive increase in fiber hydrophobicity, reaching a maximum WCA of 135°. These results indicate that the produced fibers exhibited promising morphological, compositional, thermal and magnetic properties for potential future biomedical applications, such as drug delivery and magnetic hyperthermia.

## Graphical abstract

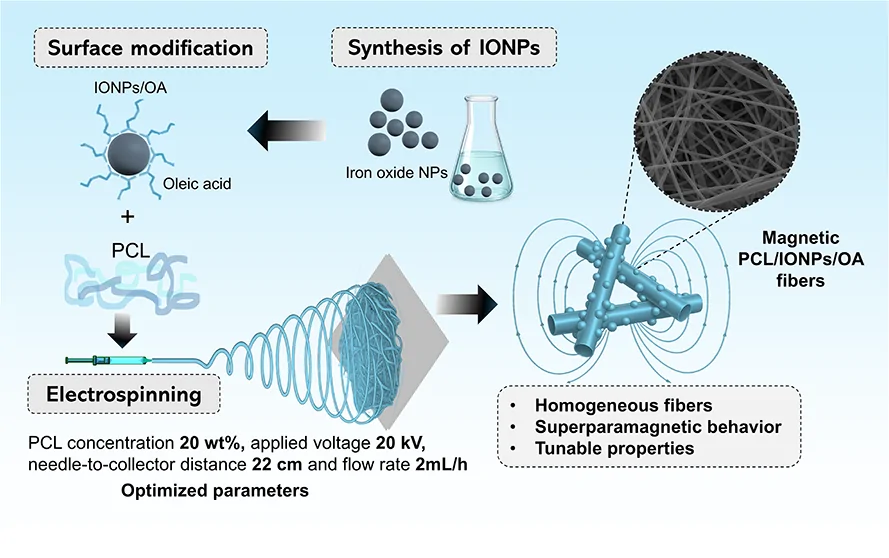

## 1 Introduction

The development of materials for biomedical applications has grown remarkably in recent years, driven by the need for more effective, selective, and minimally invasive strategies for the diagnosis and treatment of several diseases.^1,2^ In this context, electrospun polymeric fibers have emerged as promising materials due to their high surface-area-to-volume ratio, interconnected porous structure, tunable morphology and biological compatibility.^3,4^ These characteristics make them attractive for a wide range of biomedical applications, including drug delivery, tissue engineering, and antimicrobial wound dressings.^5–8^

Electrospinning is one of the most widely used techniques for producing micro- and nanofibers. This technique relies on the application of an electric field to a polymer solution, which induces the formation of a charged jet that is stretched and solidified before reaching the collector.^9^ The morphology and final properties of electrospun fibers depend on several parameters, including solution properties, such as viscosity, polymer molecular weight, solvent volatility, surface tension and electrical conductivity; processing parameters, such as applied voltage, needle-to-collector distance and flow rate; and environmental parameters, such as temperature and relative humidity.^10^

Several natural and synthetic polymers have been used to produce biocompatible fibers by electrospinning, including chitosan, polylactic acid (PLA), poly­(vinyl alcohol) (PVA), polyethylene glycol (PEG), polyvinylpyrrolidone (PVP) and polycaprolactone (PCL).^3^ Specifically, among them, PCL is a synthetic semicrystalline aliphatic polyester widely used in the biomedical field and approved by the Food and Drug Administration (FDA) for specific clinical applications.^11,12^ PCL exhibits good solubility in several organic solvents,^11^ slow hydrolytic degradation rate, excellent biocompatibility and biodegradability, and high compatibility with other biomaterials.^13^ Moreover, the mechanical response of PCL-based structures can be tailored through their morphology, porosity, composition, and processing conditions, enabling the development of materials suitable for applications such as scaffolds,^14^ implants,^15^ and polymeric membranes.^14^

The functional properties of PCL fibers can be tailored through the incorporation of nanoparticles (NPs). For instance, it was reported that the inclusion of activated carbon nanofiber nanoparticles (ACNF NPs) into PCL fibers promoted cell proliferation, with positive effects on skin tissue regeneration.^15^ The incorporation of silver (Ag) NPs into PVA/PCL fibers has also been reported to promote high antibacterial activity, achieving 100% bacterial inhibition against both Gram-positive (*S. aureus*) and Gram-negative (*E. coli*) strains.^16^ Similarly, the incorporation of magnesium oxide (MgO) NPs into PCL fibers was reported to accelerate the hydrolytic degradation of the PCL matrix during immersion in phosphate-buffered saline (PBS) at 37 °C. This behavior was accompanied by an increase in the pH of the medium, which the authors associated with the release of Mg-containing species and their neutralizing effect on the acidic degradation products generated during PCL hydrolysis. The presence of MgO NPs also promoted the precipitation of calcium phosphate apatite on the fiber surface after immersion in simulated body fluid (SBF), particularly at higher nanoparticle contents.^17^

In this context, the incorporation of iron oxide NPs (IONPs), specifically magnetite (Fe_3_O_4_), has emerged as an innovative strategy to impart magnetic properties to polymeric fibers, opening new pathways for advanced biomedical applications.^5^ Fe_3_O_4_ NPs have been extensively investigated in biomedicine owing to their magnetic properties and high biocompatibility.^18^ At the nanoscale, and specifically below a critical size (Dc), these NPs exhibit superparamagnetic behavior, characterized by the absence of remanent magnetization, low coercive field and high magnetic susceptibility.^19^ These properties facilitate their manipulation by low-intensity external magnetic fields, which are ideal for medical applications. Moreover, under an external magnetic field, these NPs can dissipate energy as heat, generating a localized temperature increase. These features make them highly suitable for applications in controlled drug delivery, anticancer therapies, magnetic resonance imaging and magnetic hyperthermia.^19–21^

However, incorporating magnetic NPs into polymeric fibers without compromising their structural uniformity requires careful optimization of the electrospinning parameters, as well as appropriate surface modification of the NPs to enhance chemical compatibility with the polymeric matrix. These practices are frequently absent in most studies reported in the literature.^22–24^ Inadequate control over these factors can lead to nonuniform magnetic and thermal responses,^25^ increased cytotoxicity of the composite, risking adverse biological reactions and ultimately compromising the material’s final performance.^26,27^ For instance, Iannotti et al. reported that the performance of electrospun magnetic nanofibers is strongly influenced by the morphology, size, concentration, and dispersion state of the incorporated NPs.^25^ In addition, Johnson et al. reported that both fiber morphology and diameter are key parameters for modulating drug release and cell response, both of which are critical in tissue engineering applications.^28^ Therefore, an integrated analysis of processing conditions and filler concentration is essential for designing magnetic electrospun fibers with controlled morphology and functional response.

In this sense, the present study reports the fabrication and characterization of magnetic electrospun fibers composed of PCL and IONPs/OA in three main stages. First, the effects of PCL concentration, applied voltage, flow rate, and needle-to-collector distance on the morphology and average diameter of neat PCL fibers were systematically evaluated to establish processing conditions capable of producing continuous, homogeneous, and defect-free fibers. Second, IONPs/OA were incorporated into the PCL fibers at 5, 10, and 20 wt % relative to PCL, and their influence on fiber morphology and diameter was investigated. The OA coating was used to improve the chemical compatibility of the IONPs and facilitate their incorporation in the polymer solution. Finally, the composite fibers were evaluated in terms of structural, compositional, magnetic, thermal, and wetting properties.

## 2 Materials and Methods

Iron­(III) chloride hexahydrate (FeCl_3_·6H_2_O), ferrous sulfate heptahydrate (FeSO_4_·7H_2_O), hydrochloric acid (HCl), polycaprolactone (PCL, *M*_n_ = 40,000 g/mol), and *N*,*N*-dimethylformamide (DMF) were supplied by Sigma-Aldrich. Ammonium hydroxide (NH_4_OH, 30%), ethanol, toluene, methanol, oleic acid (OA), and chloroform (CHCl_3_) were purchased from NEON.

### 2.1 Synthesis of IONPs

IONPs were synthesized by coprecipitation of ferric and ferrous ions (Fe^3+^ and Fe^2+^) in the presence of ammonium hydroxide, according to previously reported procedures.^29,30^ In a first step, an iron salt solution was prepared by dissolving 2.16 g of FeCl_3_·6H_2_O and 1.11 g of FeSO_4_·7H_2_O in 4 mL of HCl and 6 mL of distilled water. In a separate beaker, a second solution was prepared containing 6 mL of ammonium hydroxide and 69 mL of distilled water. Both solutions were kept separately under magnetic stirring at 55 °C for a few minutes. Subsequently, the iron salt solution was added dropwise (0.3 mL/min) into the second solution, maintaining the same temperature (55 °C) for 1 h. The progressive color change to black indicated the formation of iron oxide NPs. The resulting IONPs were washed several times by magnetic separation using distilled water and subsequently dispersed in ethanol. The general procedure is illustrated in Figure 1.

**Figure 1:**
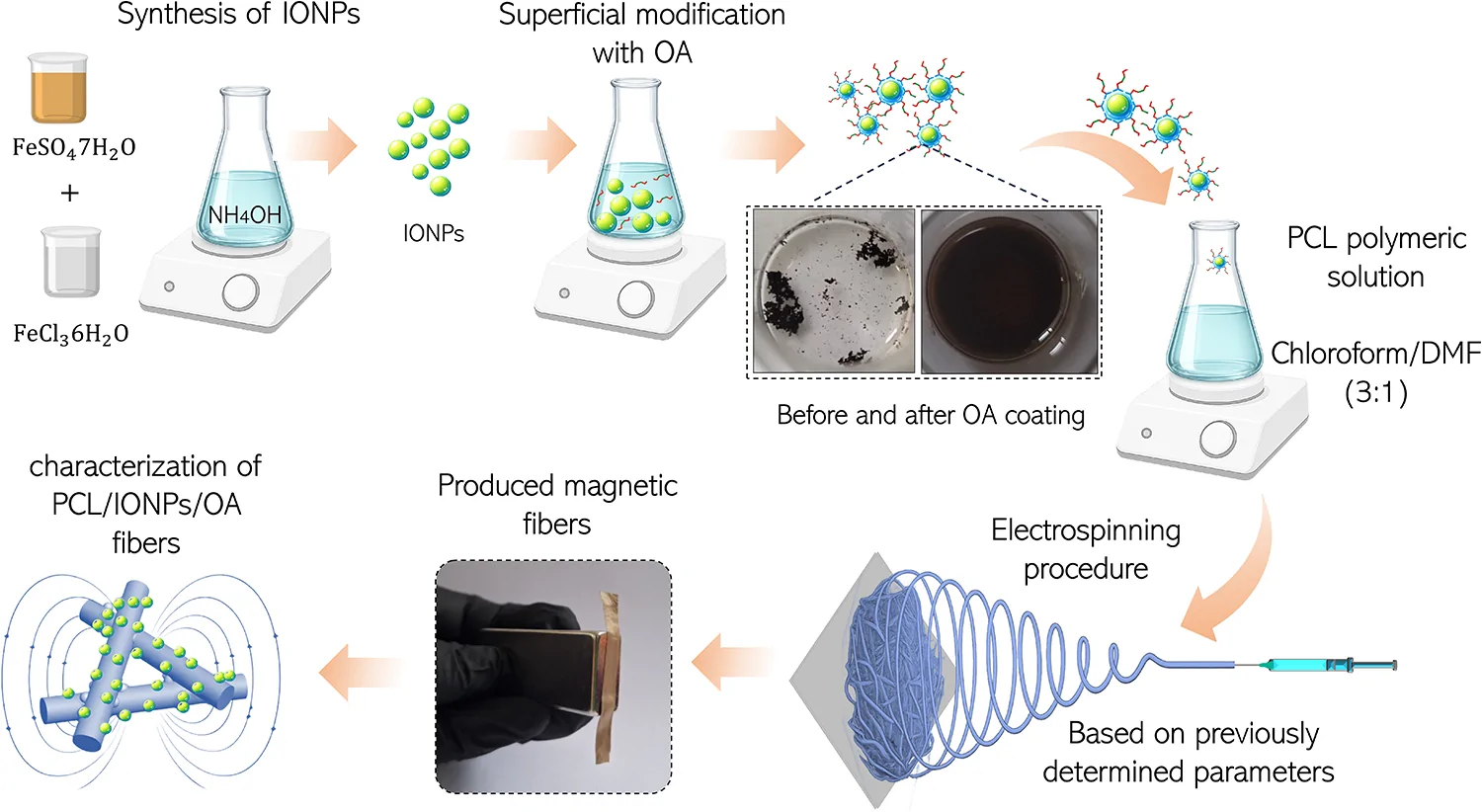
Schematic representation of the fabrication process of PCL/IONPs/OA composite fibers.

### 2.2 Surface Modification with OA

To improve the chemical compatibility of IONPs with the polymeric solution, surface modification with OA was performed. For this procedure, 0.6 g of the IONPs were redispersed in 30 mL of methanol under magnetic stirring. Subsequently, 300 μL of OA was added and ultrasonicated for several minutes. The mixture was then magnetically stirred for 2 h. After the reaction was completed, the coated IONPs/OA were washed several times by magnetic separation using toluene and finally redispersed in chloroform.

### 2.3 Preparation of Electrospinning Solutions and Fiber Fabrication

Fibers production by electrospinning was carried out based on previously reported works, with several modifications.^31,32^ In the first stage, PCL solutions at different concentrations were prepared by dissolving PCL in a chloroform/DMF mixture (3:1, v/v) for 2 h at room temperature under magnetic stirring. After selecting the most suitable PCL concentration, the applied voltage, flow rate, and needle-to-collector distance were systematically investigated using the values summarized in Table 1. After determining the conditions required to obtain homogeneous and uniform neat PCL fibers, different IONPs/OA concentrations (5, 10, and 20 wt % relative to PCL) were incorporated into fibers. For this procedure, a specific amount of IONPs/OA was first dispersed in chloroform. Subsequently, 1.5 mL of DMF was added to the mixture and magnetically stirred for 5 min. Then, 1 g of PCL was added to the solution and magnetically stirred for 1 h and 15 min. The solutions with different IONP/OA contents were sonicated for 2 min to assist homogenization of the polymer suspension before electrospinning in the polymer solution.

**Table 1:** Electrospinning Processing Parameters Employed in This Study

Parameter	Tested values
PCL concentration (%, w/v)	10, 15, and 20
Applied voltage (kV)	15, 18, and 20
Flow rate (mL/h)	0.5, 1, and 2
Needle-to-collector distance (cm)	15, 18, 22, and 26

After preparation, the PCL/IONPs/OA solution was loaded into a 5 mL syringe (equipped with a 0.8 mm diameter needle) connected to a syringe pump model Harvard Apparatus 11 Plus. Subsequently, different voltages were applied using Instor Projects and Robotics Equipment high-voltage power supply. Finally, the fibers were collected on a circular metallic collector with a radius of 5 cm, covered with aluminum foil. The electrospinning process was carried out under controlled environmental conditions, with a relative humidity of 60% ± 6% and a temperature of 24 °C ± 3 °C.

### 2.4 Characterization of Neat PCL Fibers and PCL/IONPs/OA Fibers

Fiber morphology was characterized by scanning electron microscopy (SEM) using a Philips XL30 instrument operated at an accelerating voltage of 10 kV, at magnifications of 1kx and 10kx. The samples were mounted on sample holders using double-sided carbon tape and subsequently coated with gold. Fiber diameter analyses were performed using ImageJ software. Average fiber diameters were determined from 100 randomly selected fibers for each sample. Histograms were generated using a fixed bin width of 70 nm, with a bin number of 20 for all samples, based on the number of measurements per sample and the overall diameter range analyzed (0–1400 nm). The fiber diameter data were statistically analyzed by directly calculating the mean diameter and standard deviation from 100 individual measurements per sample. The equipment was fitted with a coupled energy-dispersive X-ray spectroscopy (EDX) detector, which enabled characterization of the samples’ elemental composition.

Structural characterization of IONPs, IONPs/OA, PCL fibers, and composite fibers, was performed using a Rigaku MiniFlex 600 X-ray diffractometer (XRD) over a 2θ angle range of 10°–80° with a step size of 0.01°. Crystalline phase indexing was carried out using the Crystallography Open Database (COD) and HighScore Plus software. Chemical characterization was performed by attenuated total reflectance Fourier-transform infrared spectroscopy (ATR-FTIR) on a Bruker TENSOR 27 spectrometer over the wavenumber range of 600–4000 cm^–1^. For IONPs and IONPs/OA samples, NPs powder was compressed into a film using a hydraulic press prior to ATR-FTIR measurement. For the electrospun PCL/IONPs/OA mats, small pieces cut from the fiber mats were placed onto the ATR crystal, with the pressure clamp applied to ensure adequate contact with the sample surface.

Magnetic characterization was carried out using a MicroSense EV9 vibrating sample magnetometer (VSM), operated at room temperature under applied magnetic fields ranging from −19500 to +19500 Oe. Thermogravimetric analysis (TGA) was performed using an STA 449 F1 Jupiter thermal analyzer (Netzsch) at a heating rate of 10 °C/min under a nitrogen flow over the temperature range 23 to 600 °C. Water contact angle (WCA) measurements were performed using a Ramé-Hart model 250 F1 goniometer, and the images were analyzed using DROPimage Advanced software.

## 3 Results and Discussion

### 3.1 Analysis of Processing Parameters for Neat PCL Fibers

Fiber morphology plays an important role in biological performance. For instance, Reid et al. reported that fiber diameter is a determining factor in cell adhesion and proliferation.^33^ Similarly, Johnson et al. showed that both fiber morphology and diameter are fundamental parameters for modulating drug release.^28^ Therefore, to obtain fibers with adequate morphology, prior optimization of the electrospinning processing parameters was carried out. The effects of polymer concentration, applied voltage, flow rate, and needle-to-collector distance on the morphology and average diameter of neat PCL fibers were investigated, as discussed in the following section.

#### 3.1.1 Effect of Concentration

Figure 2 shows the fibers obtained by varying the PCL polymer concentration, while keeping the voltage (15 kV), flow rate (2 mL/h) and needle-to-collector distance (18 cm) constant. At a PCL concentration of 10%, fibers with numerous micrometric-scale bead defects were obtained. A similar behavior was observed at 15% (w/v), although it was less pronounced. It has been reported that the presence of beads is associated with fragmentation of the polymer jet, attributed primarily to the low solution viscosity and high surface tension.^34^ In contrast, at 20% (w/v), more homogeneous and continuous fibers were obtained, with no visible defects. This result was attributed to the increase in solution viscosity, which improved jet stability during electrospinning and favored the formation of homogeneous fibers.^35^

**Figure 2:**
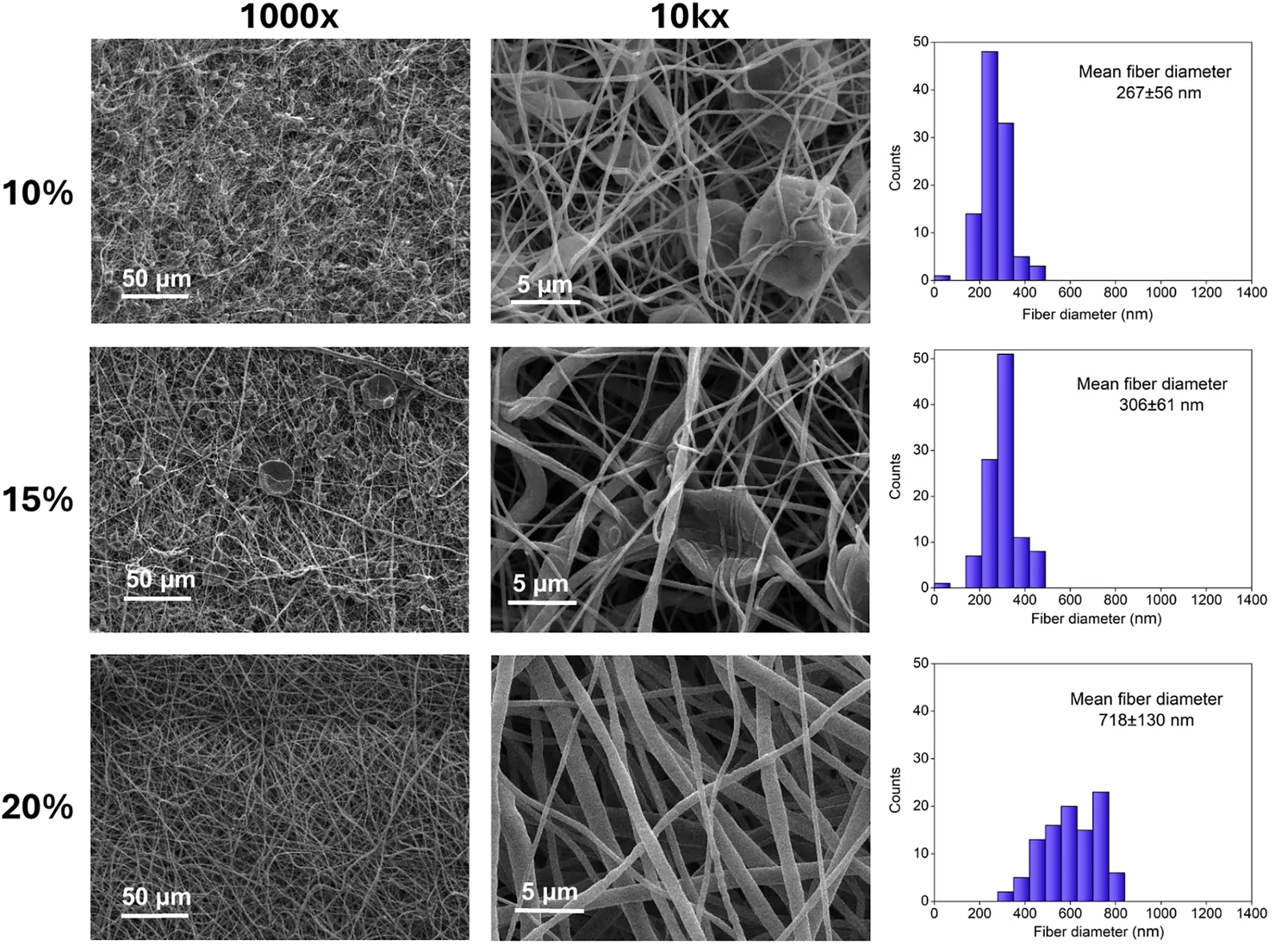
SEM images and fiber diameter distribution histograms of neat PCL fibers at different concentrations (10%, 15%, and 20%, w/v) under a fixed voltage of 15 kV, flow rate of 2 mL/h and needle-to-collector distance of 18 cm.

As shown in the histograms, increasing the PCL concentration resulted in larger average fiber diameters, reaching a maximum of 718 ± 130 nm at 20% (w/v). The increase in solution viscosity reduced the jet’s stretching capacity, resulting in fibers with larger diameters. This result is consistent with previous studies on PCL fibers, in which a progressive increase in diameter was reported as PCL concentrations increased from 5% to 10% (w/v).^36^ Based on these results, PCL concentration of 20% (w/v) was selected, as it resulted in the formation of homogeneous fibers without visible defects, which was desirable for the subsequent studies.

#### 3.1.2 Effect of Voltage

During the electrospinning process, the applied voltage determines the electric field intensity and, consequently, the charge density induced on the polymeric droplet, promoting the formation and elongation of the polymer jet toward the collector.^37^ However, jet formation only occurs when a critical voltage threshold is reached.^37^ Below this threshold, the electrostatic force is insufficient to overcome the droplet’s surface tension, restricting effective jet formation. In this study, jet formation occurred at voltages above 15 kV. Therefore, voltages of 15 kV, 18 kV and 20 kV were evaluated.

Figure 3 shows the fibers obtained at different voltages. No beads, surface defects, or apparent morphological changes were observed across all evaluated voltages. Nevertheless, a slight decrease in fiber diameter was observed with increasing voltage. This result is consistent with previous studies showing that increasing the applied voltage leads to fibers with smaller diameters due to continuous stretching caused by the electrical force.^38,39^ Based on these findings, a voltage of 20 kV was selected as the optimal condition, as the polymer jet exhibited greater stability throughout the electrospinning process.

**Figure 3:**
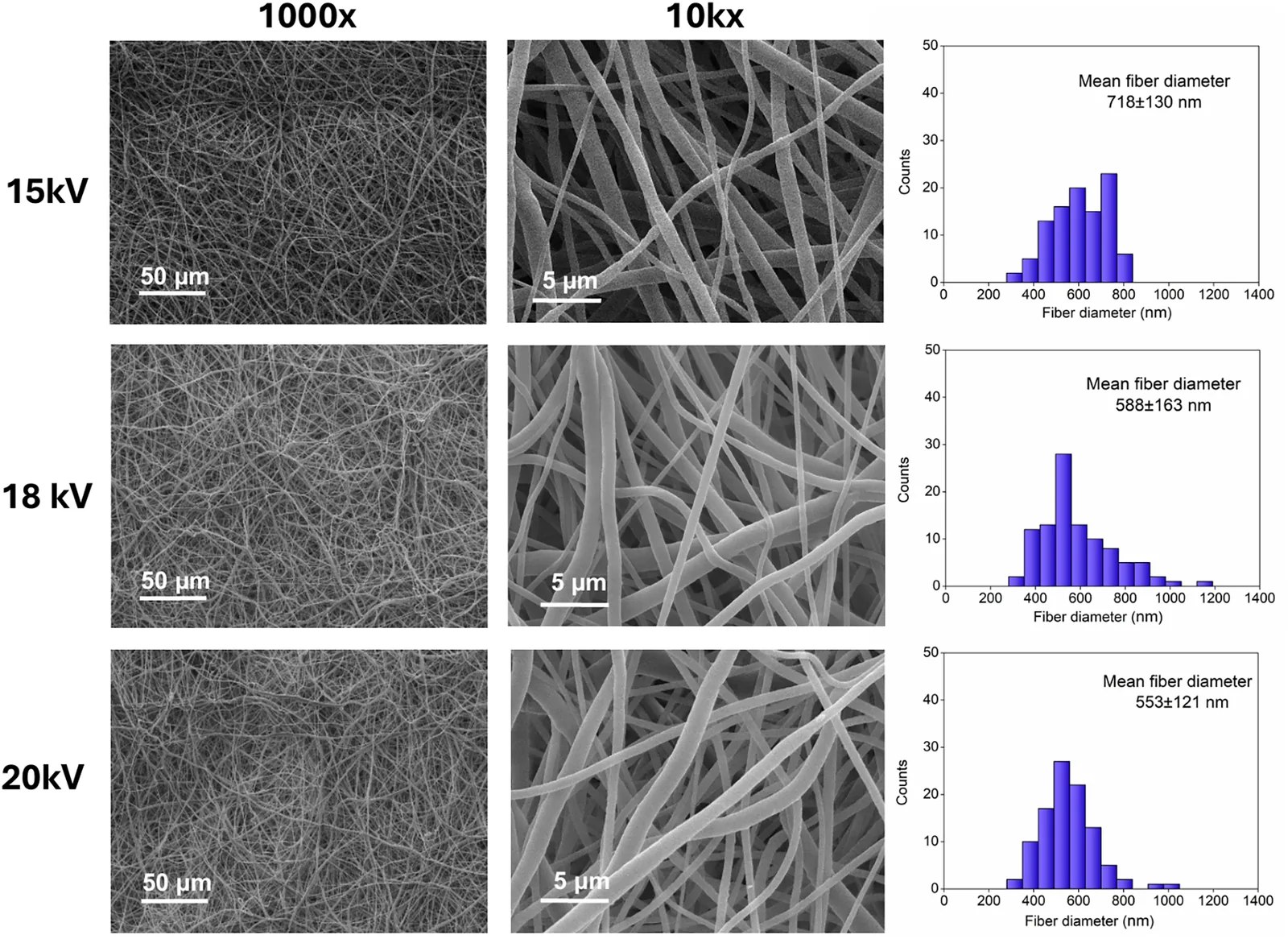
SEM images and fiber diameter distribution histograms of neat PCL fibers electrospun at different voltages (15 kV, 18 kV, and 20 kV), under a constant PCL concentration of 20% (w/v), flow rate of 2 mL/h and needle-to-collector distance of 18 cm.

#### 3.1.3 Effect of Flow Rate

The flow rate controls the amount of polymer solution expelled at the needle tip, influencing the jet formation and, consequently, the morphology and diameter of the resulting fibers.^40^ Figure 4 shows the fibers obtained at different flow rate values. At flow rates below 2 mL/h, fibers with evident interconnections and branching were obtained. This result was attributed to Taylor cone instability, likely due to an insufficient solution supply to sustain a continuous jet.^40^ At a flow rate of 0.5 mL/h, less homogeneous fibers with a more irregular morphology were observed.

**Figure 4:**
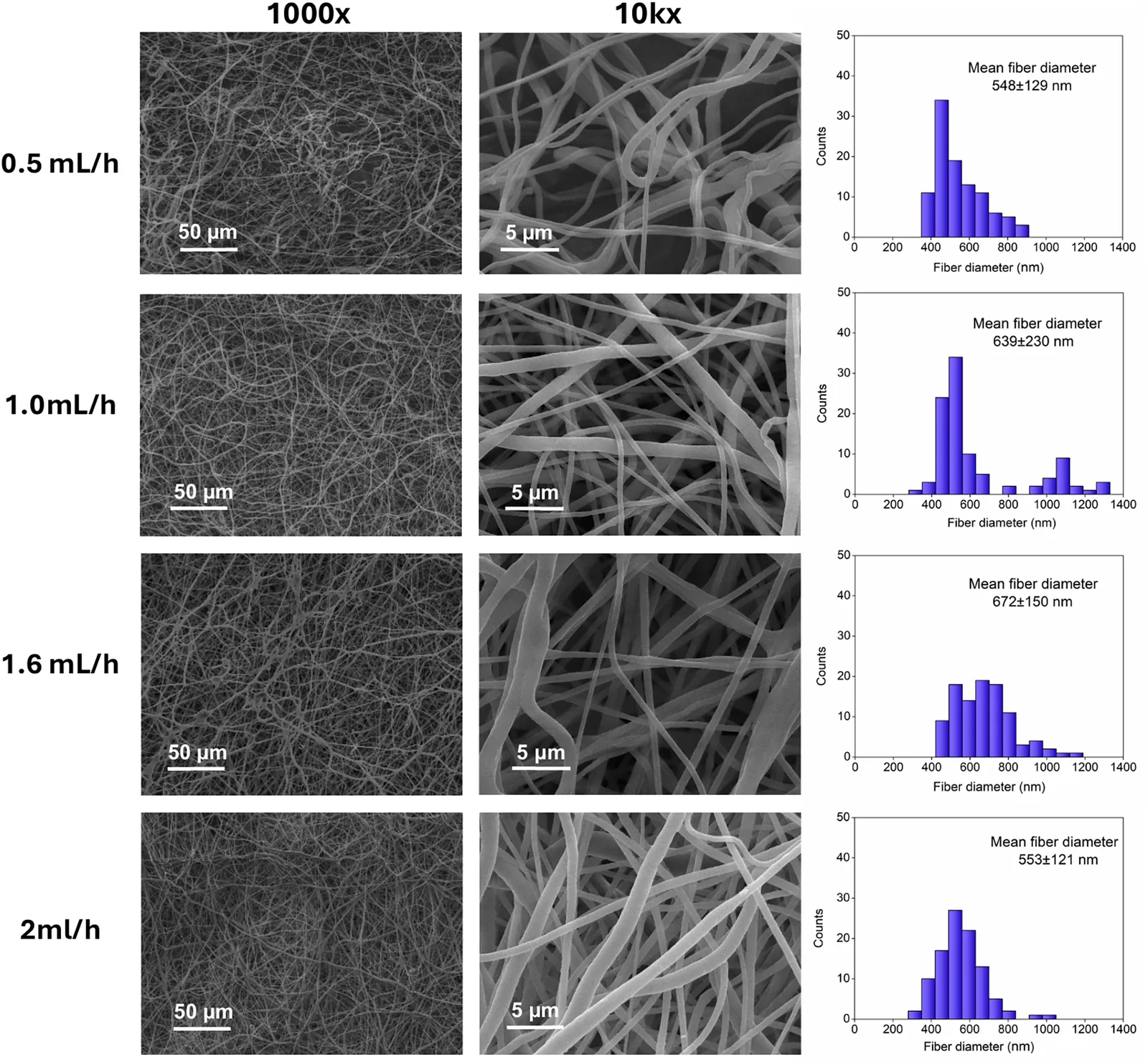
SEM images and fiber diameter distribution histograms of neat PCL fibers electrospun at different flow rates (0.5 mL/h, 1.0 mL/h, 1.6 mL/h, and 2 mL/h), under a constant PCL concentration of 20%, voltage of 20 kV, and needle-to-collector distance of 18 cm.

No significant differences in average fiber diameter were observed among the evaluated flow rates. Interestingly, this result contrasts with previous studies reported in the literature,^41,42^ which indicate that lower flow rates promote the formation of thinner and more homogeneous fibers. Based on the results, a flow rate of 2 mL/h was selected as the most suitable condition, considering that homogeneous fibers were obtained.

#### 3.1.4 Effect of Needle-to-Collector Distance

The needle-to-collector distance is a key parameter in the electrospinning process. An insufficient needle-to-collector distance can lead to incomplete solidification of the polymer jet before deposition onto the collector. In contrast, a large distance may induce jet instabilities and promote bead formation.^37^ Therefore, an optimal distance is required to allow effective solvent evaporation (chloroform/DMF, in this case), maintain jet stability, and guarantee adequate deposition onto the collector.

As shown in Figure 5, increasing the needle-to-collector distance promoted the formation of more homogeneous, oriented and thinner fibers. However, at 26 cm, morphological changes and bead formation were observed. In general, larger distances increase jet flight time, promoting more complete solvent evaporation. Nevertheless, an increase in distance can reduce the electric field intensity, causing jet instabilities and promoting bead formation,^43^ which may explain the morphological changes observed at 26 cm. Similar results were reported by Ibrahim et al., who observed an increased number of beads in PCL fibers when the needle-to-collector distance increased from 10 to 15 cm.^44^

**Figure 5:**
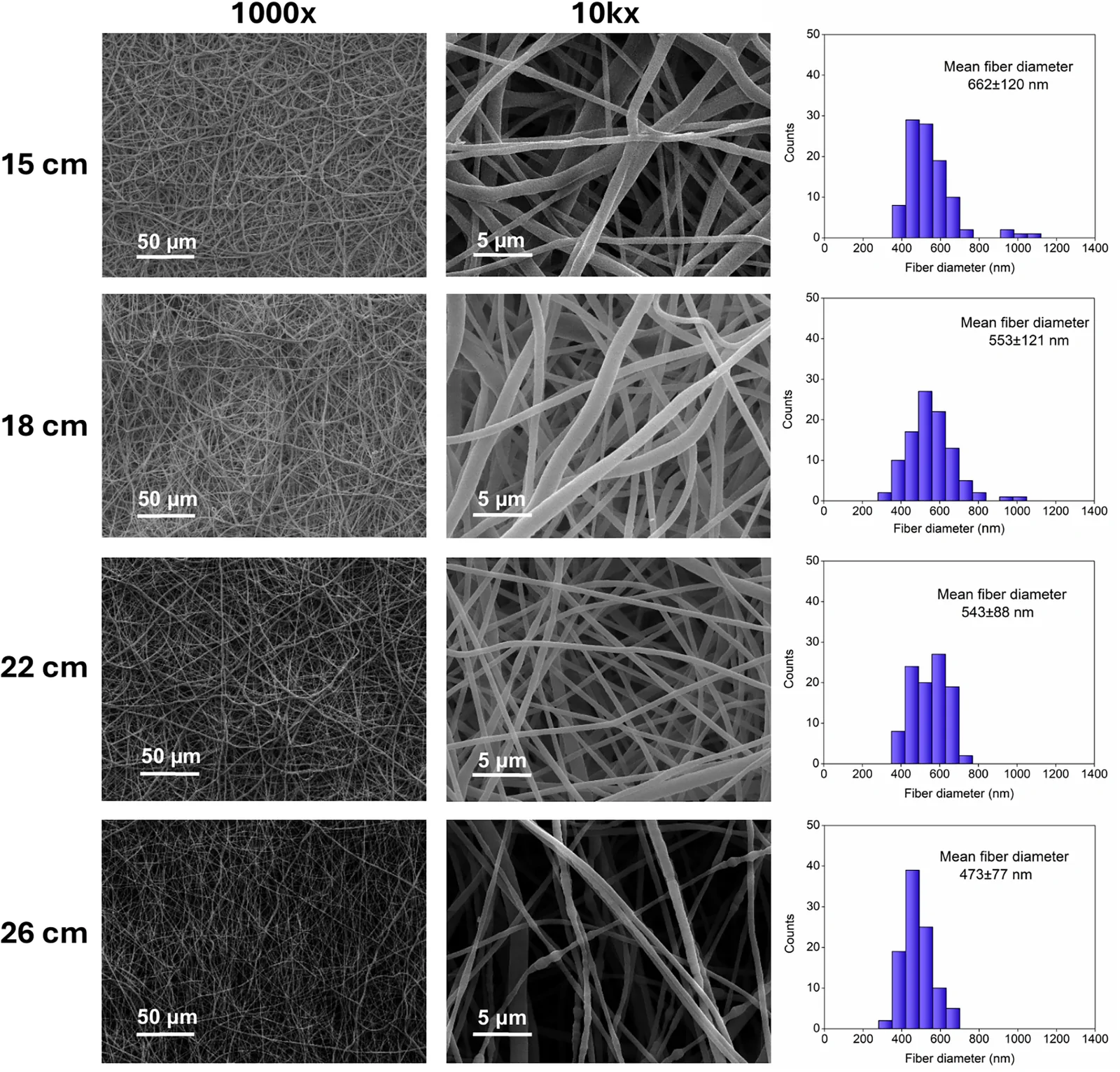
SEM images and fiber diameter distribution histograms of neat PCL fibers electrospun at different needle-to-collector distances (15, 18, 22, and 26 cm), under a constant PCL concentration of 20% (w/v), voltage of 20 kV, and flow rate of 2 mL/h.

Fiber diameter decreased as the needle-to-collector distance was increased, from 662 ± 120 nm at 15 cm to 473 ± 77 nm at 26 cm. This result could be associated with larger needle-to-collector distances, which may have allowed greater jet extension and may have contributed to the formation of thinner fibers. Based on the results, a needle-to-collector distance of 22 cm was selected as the most suitable parameter for the subsequent experiments.

Figure 6 summarizes the average fiber diameter obtained at the different PCL concentrations, voltages, flow rates, and needle-to-collector distances evaluated in this study. According to the results, the optimal electrospinning conditions selected were a PCL concentration of 20% (w/v), an applied voltage of 20 kV, a flow rate of 2 mL/h and a needle-to-collector distance of 22 cm. These conditions promoted the formation of homogeneous and uniform PCL fibers, which were desirable for subsequent studies. In addition, PCL concentration and needle-to-collector distance were the processing parameters that most strongly influenced fiber diameter.

**Figure 6:**
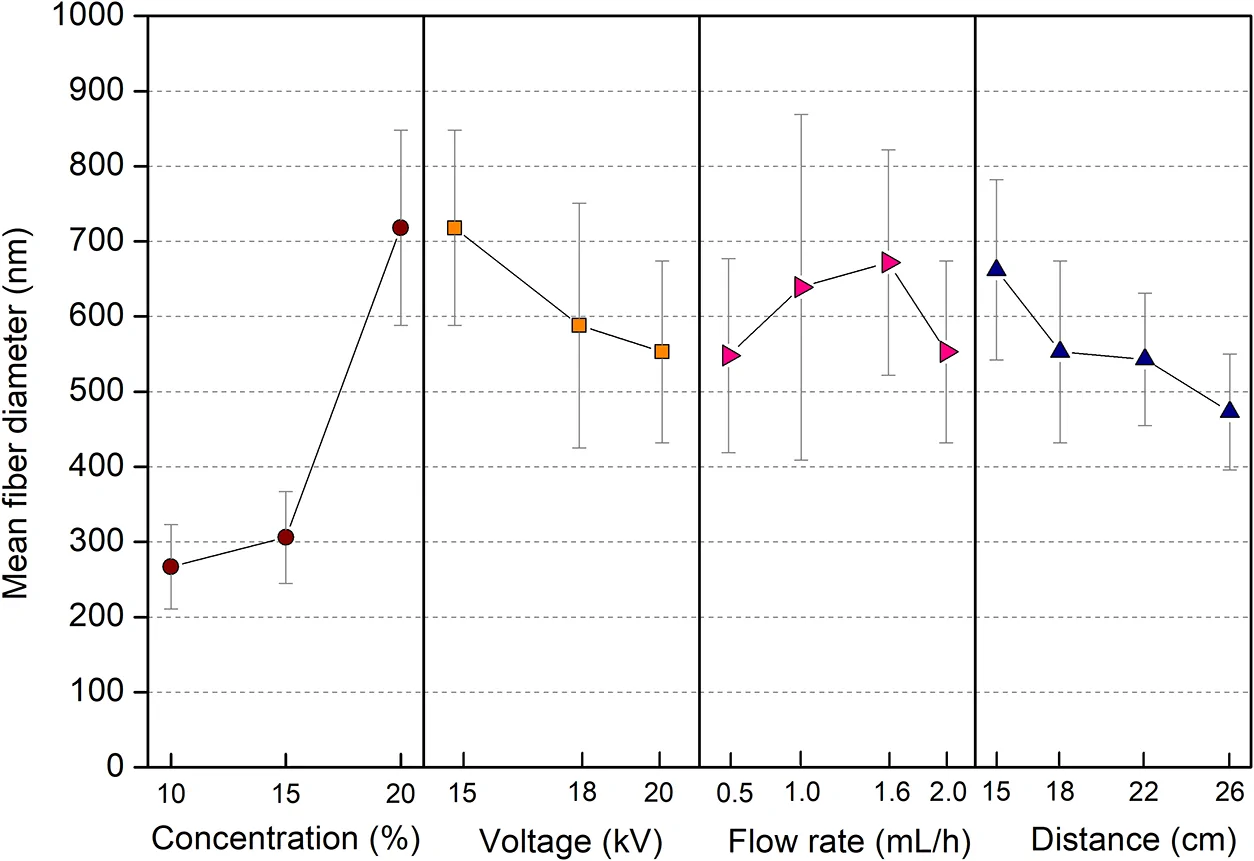
Average fiber diameter of neat PCL fibers obtained at different values of PCL concentration, voltage, flow rate, and needle-to-collector distance.

### 3.2 Characterization of PCL Fibers with IONPs/OA

After establishing the optimal processing parameters for neat PCL fiber fabrication, IONPs/OA were incorporated at concentrations of 5, 10, and 20 wt % relative to PCL, referred to as C5%, C10%, and C20%, respectively. As shown in Figure 7, the incorporation of IONPs/OA resulted in a progressive increase in fiber diameter, reaching a maximum of 961 ± 201 nm at 20 wt %. This result was attributed to an increase in the solution viscosity, which likely limited jet stretching during electrospinning and resulted in larger fiber diameters. This behavior differs from previous studies, in which higher NPs concentrations yielded smaller fiber diameters, mainly due to increased electrical conductivity of the polymer jet.^45,46^ The discrepancy suggests that, in this system, the viscosity effect may have predominated over any conductivity increase caused by the presence of NPs, thereby restricting jet elongation and favoring larger fiber diameters. Additionally, the presence of OA on the NPs’ surface may have contributed to the increase in solution viscosity, thereby limiting jet elongation.

**Figure 7:**
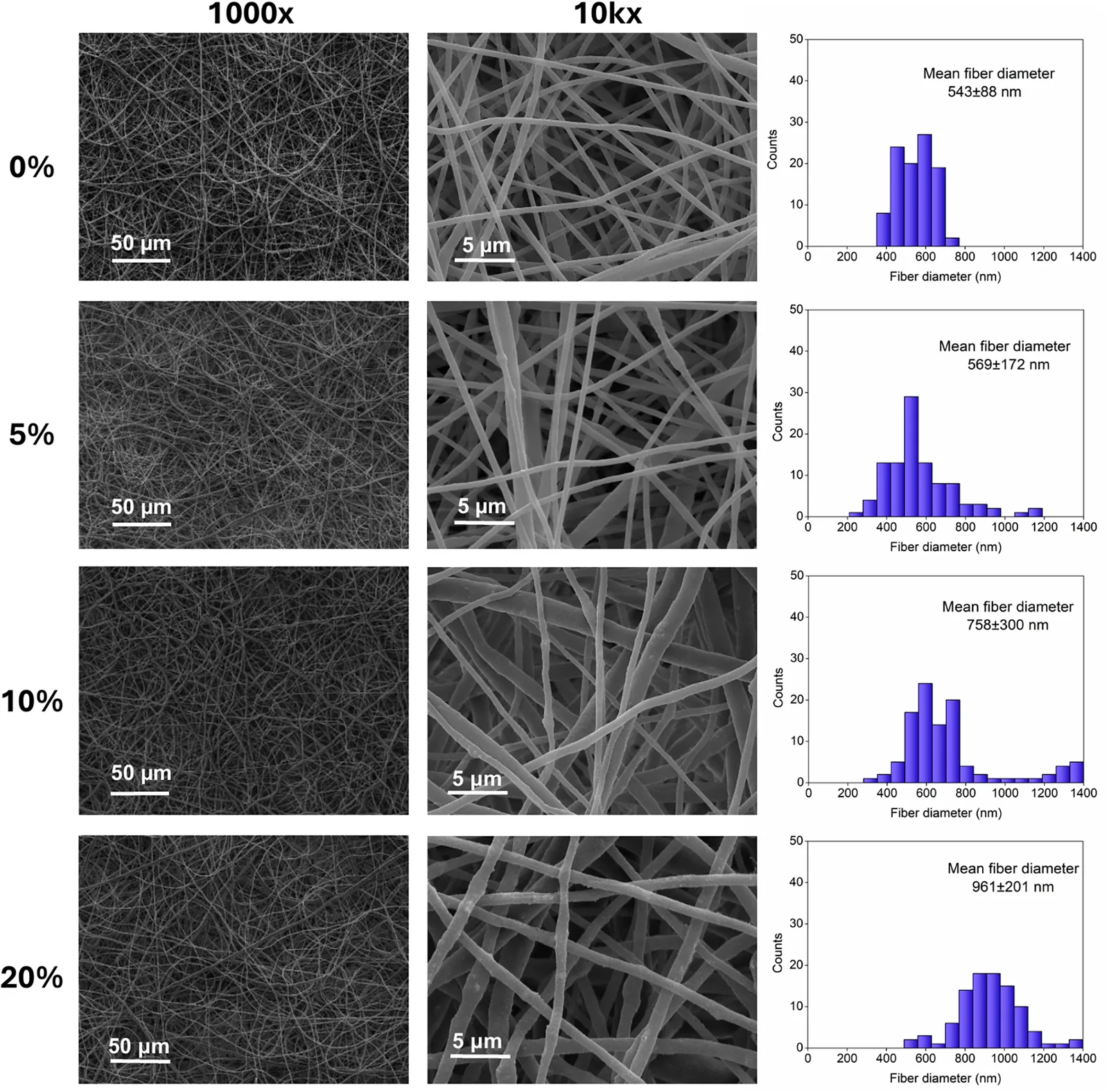
PCL fibers obtained at different IONPs/OA concentrations (0, 5, 10, and 20 wt %) using a PCL concentration of 20% (w/v), an applied voltage of 20 kV, a flow rate of 2 mL/h, and a needle-to-collector distance of 22 cm, along with the corresponding average fiber diameter distribution histograms.

#### 3.2.1 Structural and Compositional Characterization

To identify the crystalline phase of the IONPs, IONPs/OA, neat PCL fibers, composite fibers, and confirm the IONPs’ incorporation into the PCL fibers, XRD and EDX measurements were performed. The obtained diffractograms are shown in Figure 8a. The IONPs exhibited characteristic peaks at 18.2°, 30.17°, 35.3°, 37.1°, 43.1°, 53.5°, 57.2°, and 62.7°, with broadened peaks typical of materials with nanometric crystallite size.^47,48^ Based on this analysis, the IONPs showed high similarity with the magnetite phase Fe_3_O_4_ (COD ref: 96–900–2318), with characteristic planes indexed at (111), (220), (311), (222), (400), (422), (511), and (440).

**Figure 8:**
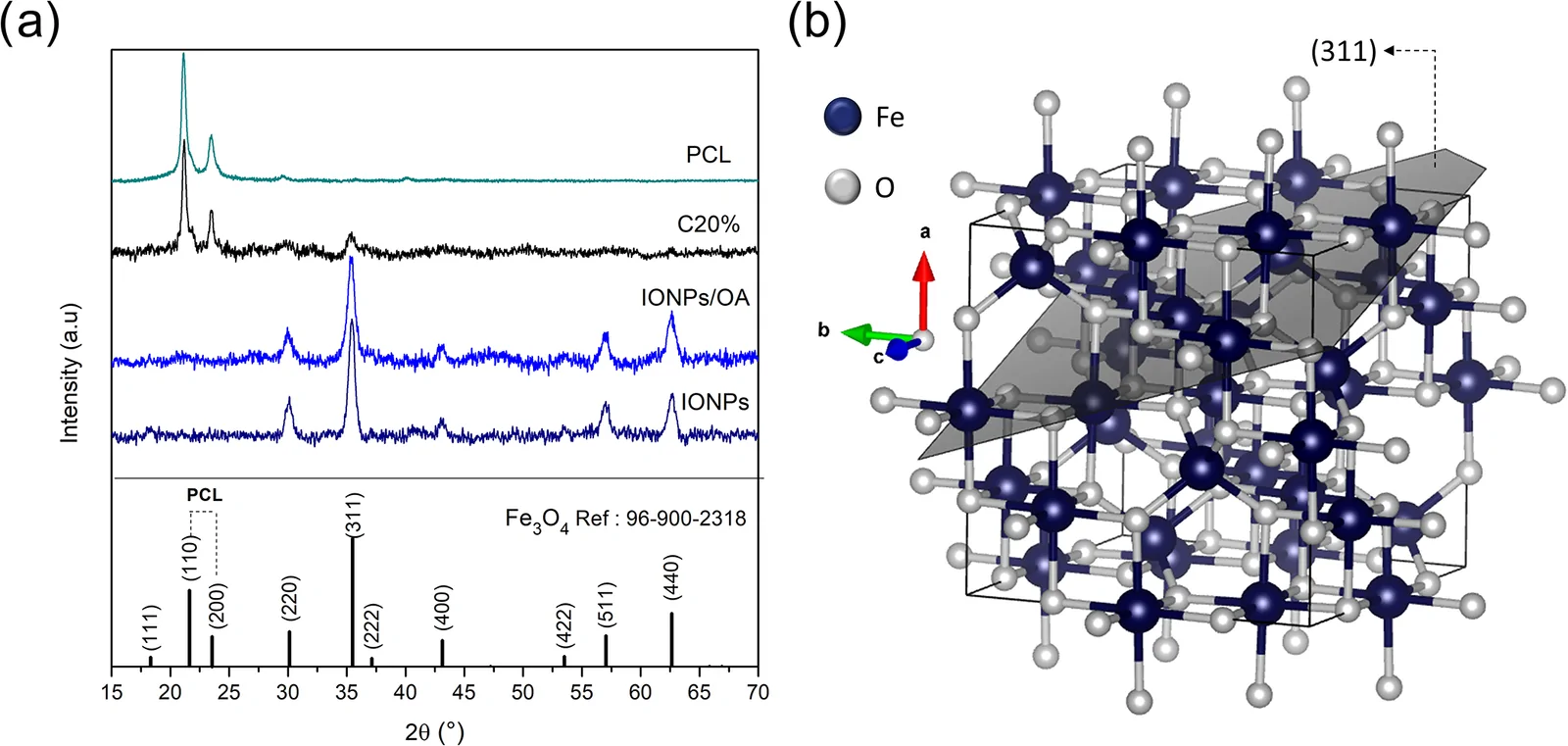
(a) X-ray diffractograms obtained for IONPs, IONPs/OA, C20% and neat PCL and (b) crystal structure phase of Fe_3_O_4_ with the (311) crystal plane highlighted.

The crystal structure of magnetite Fe_3_O_4_, obtained using VESTA software, is shown in Figure 8b. Magnetite has a spinel structure (space group *Fd*3̅*m*) with a face-centered cubic (FCC) arrangement. In this structure, Fe^2+^ and Fe^3+^ ions are distributed across tetrahedral and octahedral sites, surrounded by a close-packed arrangement of O^2–^ anions. The distribution of Fe^2+^ and Fe^3+^ ions among the crystallographic sites determines the resulting magnetic behavior of this material.^49^

The crystallite size of the IONPs was estimated from the full width at half-maximum (FWHM) of the most intense diffraction peak at 35.3° using the Scherrer equation, as described below^50^

$$D_{311}=\frac{0.89\;\lambda }{\beta \mathrm{co}s\theta }$$

where 0.89 corresponds to the shape factor, λ is the X-ray wavelength (Cu Kα, λ = 1.541874 Å), β is the peak broadening or full width at half-maximum (FWHM) of the most intense diffraction peak and θ corresponds to the Bragg angle. The average crystallite size estimated for the IONPs was 13.5 ± 2.0 nm, a value typically associated with magnetite NPs.^51–53^

For the IONPs/OA, no secondary phases were detected, confirming the preservation of the crystalline phase after OA surface coating. For neat PCL, two characteristic peaks were identified at positions of 21.5° and 23.1°, corresponding to the crystallographic planes (110) and (200), consistent with the crystallographic properties of PCL.^54^ The fibers C20% exhibited two crystalline phases attributed to the presence of IONPs/OA and PCL, confirming the incorporation of IONPs/OA into the polymeric fibers.

To identify the elemental chemical composition of the fibers, EDX measurements were performed. As shown in Figure 9, micrographs a–d, revealed the presence of C, O, and Fe with a relatively homogeneous distribution throughout the analyzed area. The micrograph in Figure 9e shows a magnified cross-sectional view of a single fiber, in which a significant Fe content could not be detected due to the equipment’s resolution limitations. Nevertheless, it can be estimated that the fibers possibly possess a low degree of surface porosity, given that no cavities or significant surface roughness were observed.

**Figure 9:**
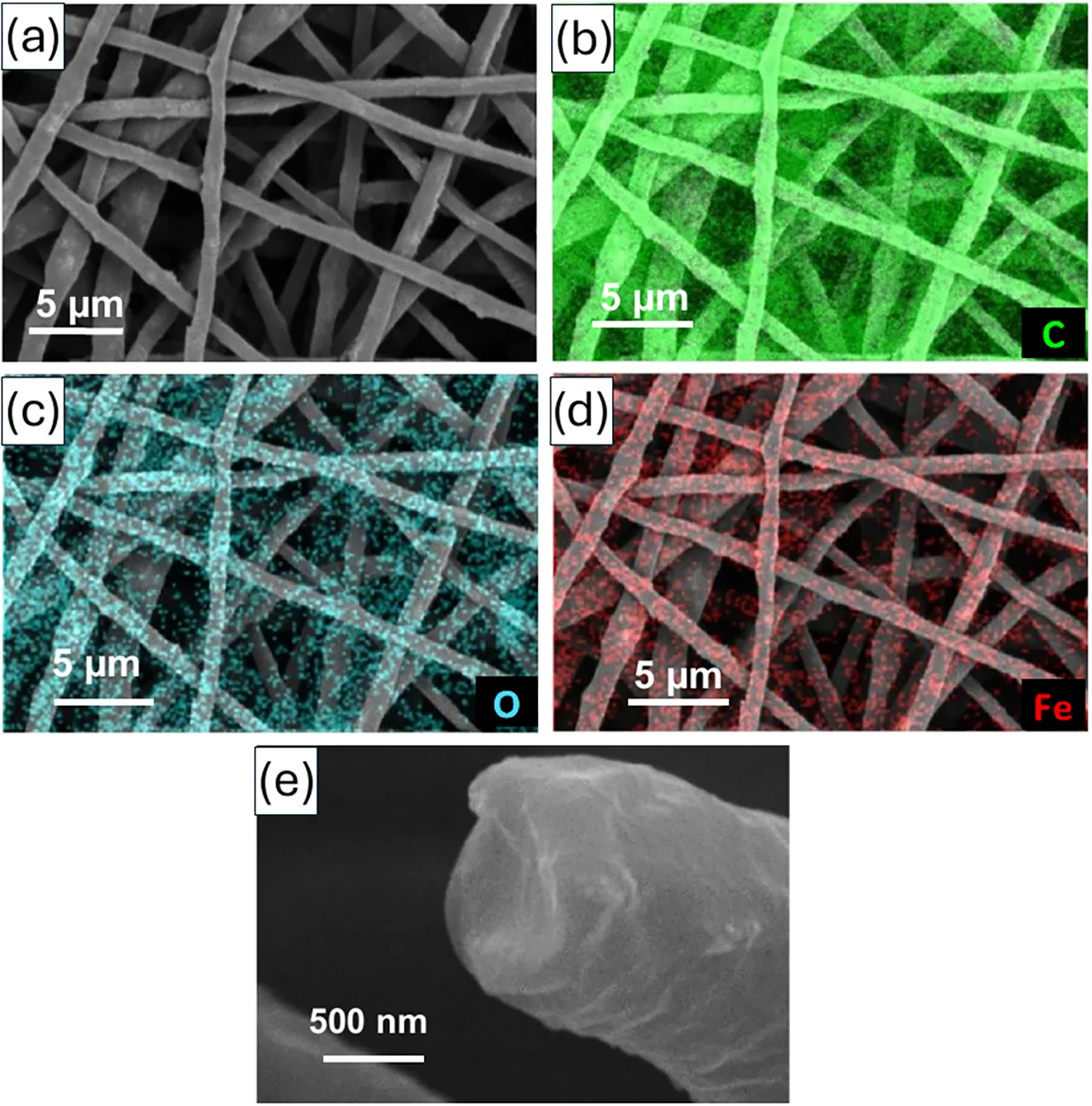
Micrographs obtained from elemental mapping by EDX analysis corresponding to C20% fibers. (a) BSE micrograph of the analyzed sample, (b) distribution of C, (c) distribution of O, (d) distribution of Fe, and (e) magnified BSE micrograph of the cross-sectional view of a single fiber.

The atomic and weight percentages obtained by EDX are presented in Table 2. For the IONPs, the atomic O/Fe ratio was approximately 1.62, slightly higher than the theoretical value of 1.33 for magnetite.^55^ This difference may be attributed to the possible oxidation of the IONPs and to the presence of water from the synthesis process. With increasing IONPs/OA concentration in the fibers, an increase in Fe content was observed. However, this increase was lower than expected based on the nominal IONP/OA content. This discrepancy may be associated with partial exclusion or loss of IONPs/OA during Taylor cone formation, jet elongation, and/or fiber collection in the electrospinning process. In addition, a decrease in the atomic percentage of carbon was observed, from 84.1 atom % to 71.1 at. %, which was attributed to the increase in the inorganic content (IONPs/OA) incorporated into the fibers.

**Table 2:** Elemental Chemical Composition Obtained from EDX Measurements for the IONPs and C5%, C10%, and C20% Fibers

Sample	Element	Weight% (wt %)	Atomic% (atom %)
IONPs	O	31.7	61.8
	Fe	68.3	38.2
C5%	C	84.1	90.4
	O	10.2	8.3
	Fe	5.7	1.3
C10%	C	80.1	88.8
	O	10.8	9.0
	Fe	9.1	2.2
C20%	C	71.1	85.1
	O	12.1	10.9
	Fe	15.6	4.0

Figure 10a shows the FTIR spectra for OA, IONPs and IONPs/OA. For the uncoated IONPs, two bands were identified at around 688 cm^–1^ and 626 cm^–1^, attributed to Fe–O stretching vibrations in the Fe_3_O_4_ crystal lattice.^56,57^ An additional band at 1626 cm^–1^ was attributed to the bending vibrations of H–O–H bonds, indicating a possible presence of water.^58^ This result may be attributed to the coprecipitation synthesis process, which was carried out in an aqueous medium. The bands at 2380 cm^–1^ and 2336 cm^–1^ were attributed to the presence of atmospheric CO_2_ during the measurement process.^59^

**Figure 10:**
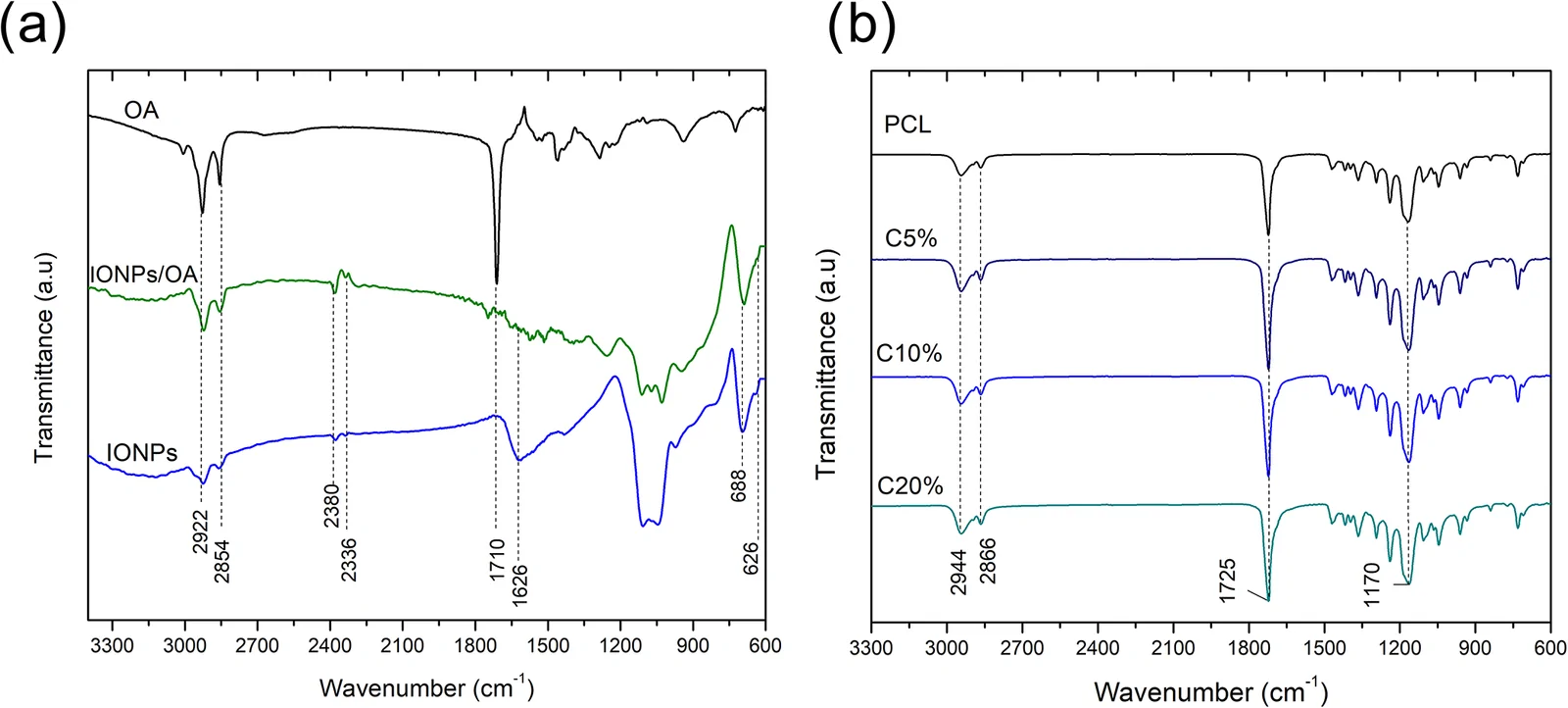
FTIR spectra obtained for (a) IONPs, IONPs/OA, and OA, and (b) neat PCL fibers, and C5%, C10%, and C20% fibers.

For the IONPs/OA, an increase in the intensity of the bands at 2922 cm^–1^ and 2854 cm^–1^ was observed. These bands were attributed to a higher presence of CH_2_ and CH_3_ groups of OA disposed on the surface of the IONPs.^60^ In the OA spectrum, a band around 1710 cm^–1^ corresponding to the stretching vibration of C=O groups was observed.^61^ However, this band was not identified in IONPs/OA. This result was probably due to the carboxyl group – COOH of OA adhering to the surface of the IONPs, forming bonds with the Fe–O metallic atoms.^62,63^

Figure 10b shows the spectra for neat PCL and the composite fibers with different IONPs/OA concentrations. Two bands were identified at 2944 cm^–1^ and 2866 cm^–1^, attributed to the asymmetric and symmetric stretching vibrations of the CH_2_ groups characteristic of PCL.^64^ In addition, a band at 1725 cm^–1^ was attributed to the stretching vibrations of the C=O groups present in the PCL polymeric structure. A band at around 1170 cm^–1^ was attributed to symmetric C–O stretching vibrations. The PCL bands observed were consistent with previous studies reported in the literature.^64–66^

After IONPs/OA incorporation, no significant differences were observed in the spectra compared to neat PCL. Additionally, no perceptible bands associated with IONPs/OA were identified, likely due to the relatively low amount of IONPs/OA incorporated into the fibers and to the weak spectral contribution and possible overlap of their characteristic absorption bands with the intense bands of the PCL matrix.

#### 3.2.2 Magnetic Characterization

To evaluate the magnetic behavior of the fibers, VSM measurements were performed. Figure 11 shows the magnetization curves (M) as a function of the applied magnetic field (H) obtained at room temperature (*T* = 300 K) for the different samples. As shown in Figure 11a, superparamagnetic behavior was observed at room temperature, with saturation magnetization *M*_s_ values of 42.6 ± 0.4 emu/g and 40.5 ± 0.4 emu/g for the IONPs and IONPs/OA, respectively (Table 3). These values are consistent with *M*_s_ values reported in the literature for magnetite NPs.^67^ In addition, no significant differences in coercivity (*H*_c_) values were observed between the samples. The superparamagnetic behavior obtained was considered desirable, since a high magnetic response is achieved under low-intensity magnetic fields, which could be advantageous for biomedical applications.

**Figure 11:**
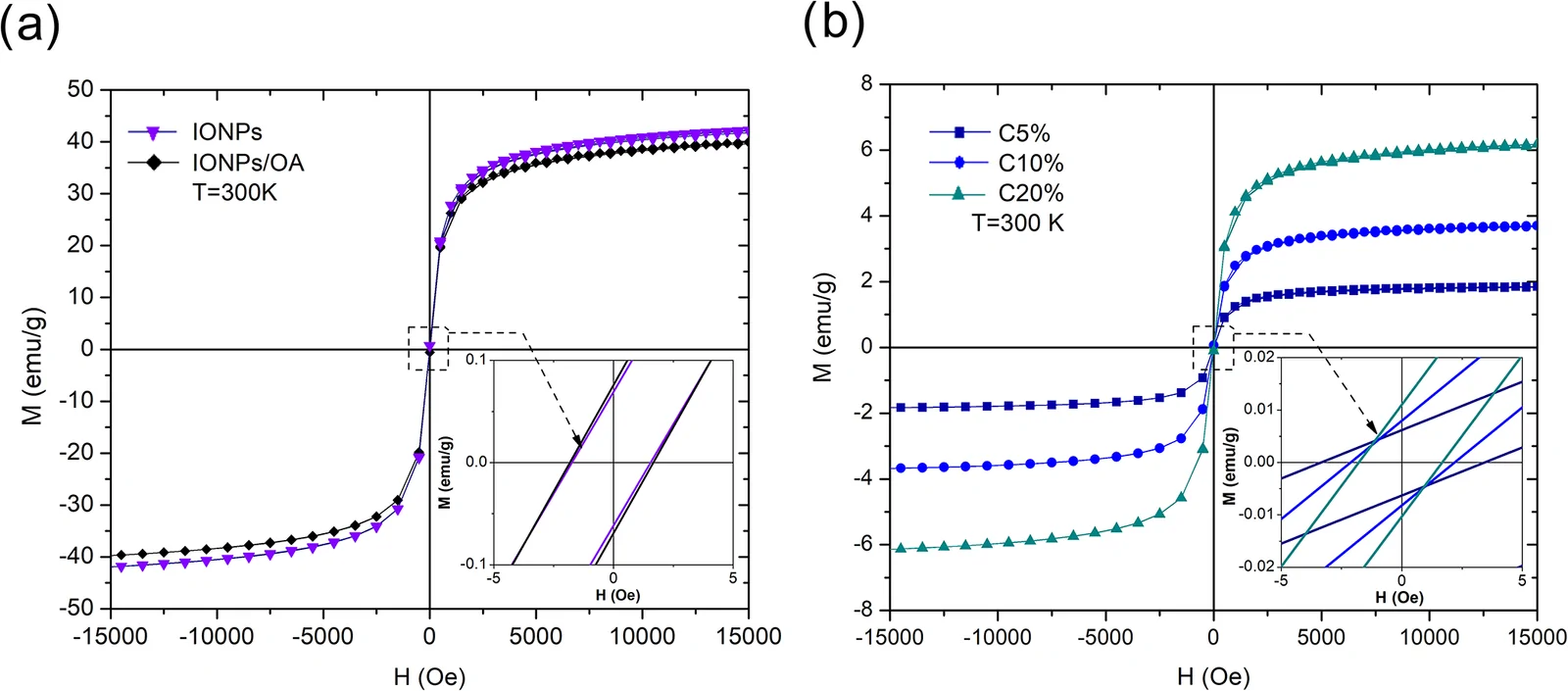
VSM measurements obtained for (a) IONPs and IONPs/OA, and (b) C5%, C10%, and C20% fibers, performed at a temperature of 300 K.

**Table 3:** Magnetization Values Obtained from VSM Measurements for the Fibers at Room Temperature^a^

Sample	*M*_s_ (emu/g)	*H*_c_ (Oe)
IONPs	42.6 ± 0.4	1.7 ± 0.1
IONPs/OA	40.5 ± 0.4	1.8 ± 0.1
C5%	1.8 ± 0.1	1.7 ± 0.1
C10%	3.6 ± 0.1	2.2 ± 0.1
C20%	6.1 ± 0.1	3.3 ± 0.1

a *M*_s_ denotes the saturation magnetization of the samples in emu/g, and H_c_ denotes the coercive field in Oe.

Interestingly, the OA coating had only a minimal influence on the *M*_s_ of the IONPs compared with other commonly used organic coating agents, such as polyethylene glycol (PEG) and chitosan, which generally produce a significant reduction in *M*_s_ of approximately 20–30 emu/g.^68–70^ These results suggest that surface modification with OA is a viable strategy to promote chemical compatibility in organic media without significantly compromising the magnetic properties of the NPs.

As shown in Figure 11b, the incorporation of IONPs/OA yielded fibers with superparamagnetic behavior at room temperature and low *H*_c_ values. The *M*_s_ showed a significant decrease compared to IONPs/OA, reaching a maximum value of 6.1 ± 0.1 emu/g for C20% fibers. This decrease was attributed to the presence of nonmagnetic components associated with PCL.^71^ Despite this significant reduction in *M*_s_, the values obtained were comparable to those reported in the literature (ranging from 1.87 emu/g to 10 emu/g).

These values have been demonstrated to be sufficient to generate a functional magnetic response capable of promoting controlled drug release, inducing apoptotic calorific effects in cancer cells, supporting tissue regeneration and even driving cellular differentiation.^72–74^ Finally, the magnetic response tendency was consistent with the amount of IONPs/OA incorporated into the fibers.

#### 3.2.3 Thermogravimetric Characterization

TGA measurements were performed to evaluate changes in the thermal degradation behavior of PCL induced by the presence of the IONPs within the fibers and estimate, through the residual mass, the inorganic IONPs fraction retained in the composite fibers after degradation of the organic components. Figure 12 shows the TGA and DTG curves obtained for neat PCL and composite fibers, and Table 4 summarizes the corresponding values. Neat PCL exhibited a single thermal degradation stage at around 405.8 °C, attributed to the depolymerization of PCL into ε-caprolactone.^75^ At temperatures above 400 °C, a complete degradation of the material occurred, resulting in a residual mass of 0% at 500 °C.

**Figure 12:**
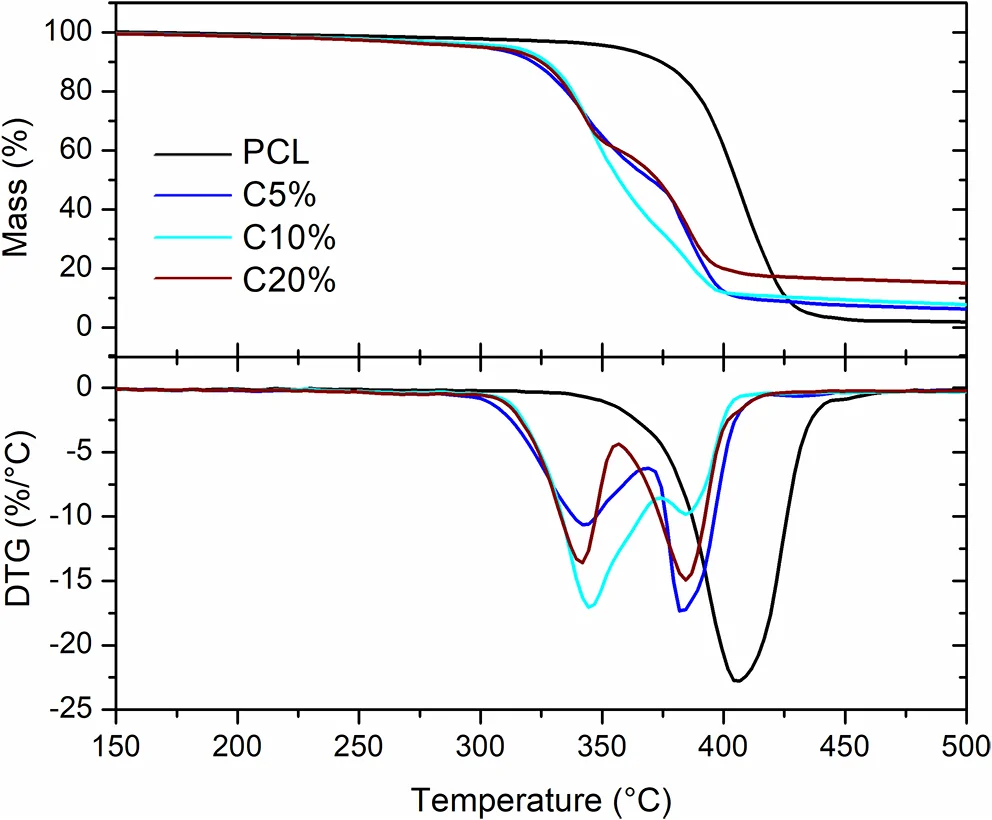
TGA and DTG results for neat PCL and C5%, C10%, and C20%.

**Table 4:** Degradation Peaks and Residual Mass Obtained from TGA Measurements for C5%, C10% and C20%

Sample	1st Peak weight lost [°C]	2nd Peak weight lost [°C]	Residual mass[%]
PCL	405.8	-	0
C5%	342.4	383.7	2.6
C10%	341.8	384.6	4.7
C20%	345.5	382.8	10.7

Upon incorporation of different IONPs/OA contents, two distinct thermal degradation stages were observed between 340 and 400 °C. This behavior was likely due to random PCL pyrolysis promoted by the presence of IONPs.^7^ This effect may have altered the thermal stability of different segments of the PCL polymer chains, promoting the separation of the degradation events and shifting the degradation maxima toward lower temperatures. Similar behavior has been reported previously for polymer composites incorporating NPs into PCL, indicating that the presence of NPs modifies the thermal degradation profile of PCL.^76,77^ Additionally, the degradation stage observed between 340 °C–347 °C may have included an overlapping contribution from the decomposition of OA bound present on the surface of the IONPs, particularly through rupture of its hydrocarbon chain (C–C).^55^ The thermal decomposition of OA has been reported within a similar temperature range, particularly when OA is bound to the surface of IONPs.^78,62,55^ Additionally, the C10% sample exhibited a slightly different thermal profile compared with C5% and C20%. This difference may be related to variations in the relative contributions of overlapping PCL and OA degradation processes, as well as possible differences in NP dispersion and agglomeration compared to the other composites. However, the temperatures of the two degradation maxima remained similar among the composites.

Neat PCL fibers exhibited negligible residual mass at 500 °C, with a value close to 0% within the experimental resolution. Taking this value as a reference, the residual masses of 2.6%, 4.7%, and 10.7% obtained for C5%, C10%, and C20%, respectively, were used to estimate the inorganic IONPs fraction retained in each composite after degradation of the organic components. The residual mass values were lower than the nominal IONPs/OA contents, a result similar to that obtained by EDX, which may be attributed to a nonhomogeneous distribution of IONPs/OA within the fibers and possible material loss during electrospinning. Such differences are frequently reported in the literature and are generally ascribed to experimental and measurement-related factors.^79^

#### 3.2.4 Wettability Evaluation

The hydrophobicity of a material is an important property for biomedical applications, because it governs interactions between the material surface and the biological environment, directly influencing processes such as protein adsorption, cell adhesion and proliferation, and autoimmune responses.^80,81^ The degree of surface hydrophobicity can be estimated through WCA

of IONPs/OA. This behavior may be mainly associated with the hydrophobic character of the OA-coated IONPs, as well as with the possible local morphological heterogeneity induced by measurements, where high angle values (θ > 90°) indicate high surface hydrophobicity.^82^

Figure 13 shows the WCA obtained for neat PCL fibers and composite fibers. For PCL fibers without IONPs/OA content, a contact angle of 119° ± 2° was obtained, consistent with the hydrophobic property of PCL, primarily associated with the presence of CH_2_ functional groups in the polymer chain.^83,84^ With increasing IONPs/OA content, a progressive increase in the contact angle was observed, yielding values of 125° ± 1°, 131° ± 1°, and 134° ± 2° for C5%, C10%, and C20% fibers, respectively. This result indicated an increase in the hydrophobicity of the fibers upon incorporation of IONPs/OA. This behavior may be mainly associated with the hydrophobic character of the OA-coated IONPs, as well as with the possible local morphological heterogeneity induced by NP incorporation, which may promote a Cassie–Baxter wetting state.^85^ Previous studies have shown an increase in the surface roughness of PCL fibers as the NP concentration increased, leading to a progressive increase in WCA compared to neat PCL fibers.^86,87^ However, quantitative surface roughness measurements would be necessary to determine whether this effect contributed to the increase in WCA observed.

**Figure 13:**
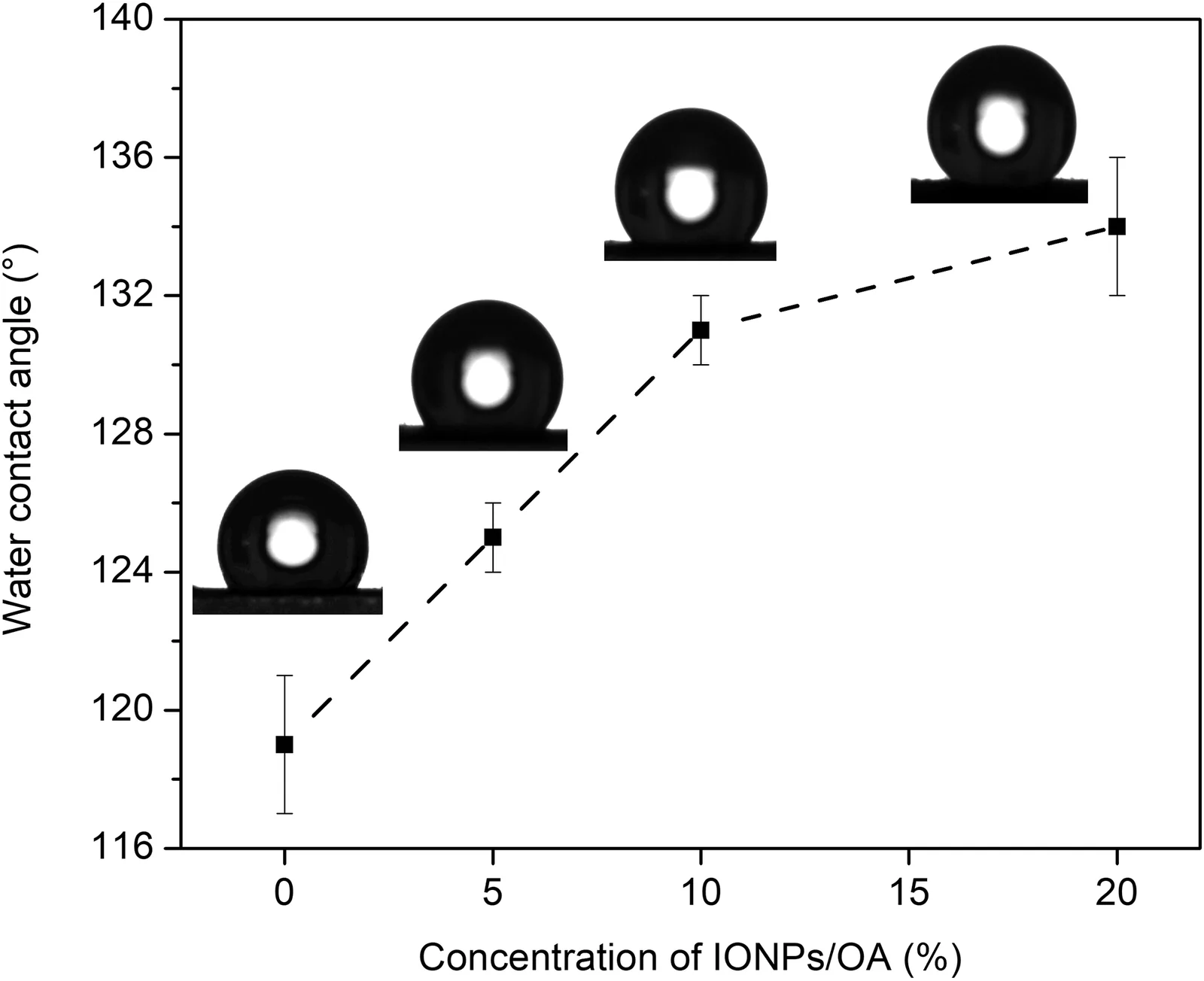
WCA for neat PCL fibers and C5%, C10% and C20% fibers.

## 4 Conclusions

In this study, magnetic polymeric fibers composed of PCL and IONPs/OA were produced by electrospinning. The processing conditions for neat PCL fiber production were a PCL concentration of 20% (w/v), an applied voltage of 20 kV, a needle-to-collector distance of 22 cm, and a flow rate of 2 mL/h, yielding homogeneous fibers with an average diameter of 543 ± 88 nm. Among the evaluated processing parameters, PCL concentration and needle-to-collector distance had the strongest influence on fiber diameter. IONPs/OA incorporation increased the average fiber diameter, reaching 961 ± 201 nm for C20% fibers.

Magnetic measurements confirmed that the composite fibers exhibited superparamagnetic behavior at room temperature, with a maximum *M*_s_ of 6.1 ± 0.1 emu/g for C20% fibers, representing a promising value for magnetically responsive polymeric fibers. In addition, the OA coating had minimal influence on the *M*_s_ of the IONPs, indicating that OA is a viable coating strategy to improve their chemical compatibility with PCL without significantly compromising magnetic properties. The results also demonstrated that IONPs/OA incorporation modified the thermal and wetting responses of the fibers. TGA revealed two degradation events in the composite fibers and a shift of PCL degradation toward lower temperatures following the incorporation of IONPs/OA. Furthermore, IONPs/OA incorporation led to a progressive increase in WCA, reaching approximately 135° for C20% fibers, indicating increased hydrophobicity.

This work integrates electrospinning conditions and filler concentration to produce homogeneous PCL/IONP/OA fibers with promising morphological, compositional, thermal, and magnetic properties for potential future biomedical applications, such as drug delivery and magnetic hyperthermia. However, further biocompatibility studies, such as cell viability assays, are required to assess their potential suitability for biomedical applications.
